# The metabolic implications of maternal exercise: effects on pregnant women and their offspring

**DOI:** 10.3389/fnut.2026.1741714

**Published:** 2026-02-19

**Authors:** Zhenglin He, Yutong Yuan, Lingkai Zhang, Wentong Niu, Liyu Liu, Baoer Chen, Xue Wang

**Affiliations:** 1 Department of Clinical Nutrition, China-Japan Union Hospital of Jilin University, Changchun, China; 2 Department of Clinical Nutrition, First Hospital of Jilin University, Changchun, China; 3 State Key Laboratory of Female Fertility Promotion, Department of Obstetrics and Gynecology, Center for Reproductive Medicine, Peking University Third Hospital, Beijing, China; 4 Department of Clinical Nutrition, Xinhua Hospital Affiliated to Shanghai Jiao Tong University School of Medicine, Shanghai, China; 5 School of Public Health, Shanghai Jiao Tong University School of Medicine, Shanghai, China

**Keywords:** maternal exercise, metabolism, nutrient, offspring, pregnancy

## Abstract

Metabolic health is increasingly recognized as a vital issue in global health, particularly with respect to pregnant women. This population is uniquely vulnerable to metabolic disorders, such as gestational diabetes, due to lifestyle modifications and physiological changes that occur during pregnancy. Additionally, the metabolic state of pregnant women significantly impacts the metabolic health of offspring. To enhance metabolic health, proper exercise is essential. This review examines the effects of maternal exercise on the metabolic health of pregnant women and their children, highlighting the evidence linking maternal exercise to various metabolic complications and the mechanisms that underpin its benefits.

## 1 Introduction

Pregnant women represent a vulnerable population whose health concerns span two generations. Globally, over 100 million pregnant women will choose to give birth by 2025, but millions of women die every year due to pregnancy complications (1), and adverse changes in the weight and metabolic indicators can lead to the occurrence of high-risk pregnancies, with low-birth-weight or overweight infants facing future health threats. Current strategies to improve maternal health and support the well-being of unborn children predominantly focus on nutritional counseling, often leading to unnecessary supplementation, even for obese pregnant women (2). Characteristics and imbalances within metabolic processes are closely associated with the metabolism of proteins, fats, carbohydrates, and other essential nutrients (3). No less important than nutritional support during pregnancy, the implementation of appropriate exercise serves as a safe and effective non-pharmacological intervention, essential for optimizing metabolic health in both mother and fetus (4). Building on the need for comprehensive prenatal care, the imperative to integrate structured exercise into pregnancy regimens is increasingly clear. The US Department of Health and Human Services (DHHS) and the American College of Obstetricians and Gynecologists (ACOG; 2020) recommend that pregnant women without contraindications engage in exercise for at least 20–30 min per day, or a total of at least 150 min per week (5–7). However, multiple clinical studies indicate that a limited number of pregnant women meet these recommendations (8–10). Misconceptions about exercise during pregnancy persist, with some believing it may increase the risk of complications such as miscarriage (11). Nonetheless, research suggests that these concerns are unfounded, and regular exercise during pregnancy can actually reduce the risk of adverse perinatal outcomes.

Excessive weight gain and metabolic disorders during pregnancy can adversely affect offspring health as observed in animal studies (12). Numerous studies have shown that exercise during pregnancy is safe and beneficial for fetal and child health (13), although recent studies in rodents identified a potential negative association between exercise, fetal growth, and systemic obesity (14). Preclinical research indicates that the integrations of exercise and a specific dietary regimen can enhance skeletal muscle function, promote carbohydrate and lipid metabolism, regulate gut microbiota, and improve overall metabolic health (15). The benefits of exercise extend beyond the duration of activity, even allowing individuals to retain advantages even after discontinuing exercise due to sustained metabolic capacity. This lasting protection is attributed to adaptations such as increased skeletal muscle flexibility and enhanced mitochondrial function (16, 17), along with molecular mechanisms like sustained epigenetic modifications (18). Beyond its role as a beneficial adjunct, prescribed physical activity must be recognized as a critical, non-negotiable component of antenatal management.

International prenatal physical activity guidelines have shifted from general wellness promotion to evidence-based therapeutic prescriptions aimed at reducing pregnancy complications and enhancing long-term health outcomes. This evolution is reflected in recent guidelines, such as those from Canada (2019) (19) and Australia (2022) (20). A global consensus, supported by World Health Organization (WHO) and European Board and College of Obstetrics and Gynecology (EBCOG), endorses moderate-intensity exercise while recommending caution with high-intensity regimens due to physiological risks (21). For clinical conditions like gestational diabetes, combined aerobic and resistance training is specifically advised (22). In alignment with this global trend, China established a national expert consensus in 2025 to promote standardized implementation. Moving forward, key challenges include advancing individualized exercise protocols and addressing persistent translational barriers in clinical practice.

This review aims to explore the impact of maternal exercise on nutrient partitioning and energy metabolism, thereby influencing the metabolic health of both mother and offspring. To enhance these benefits, it is essential to highlight and specify key parameters such as exercise duration, frequency, and intensity grounded in safety and efficacy principles. Additionally, we also discuss recent mechanistic insights into how maternal exercise modulates placental function and fetal metabolic development, thus providing a physiological basis for its inclusion in prenatal care.

## 2 Exercise during pregnancy and maternal metabolic health

### 2.1 Maternal adaptations in nutrient metabolism during pregnancy and related outcomes

**Figure 1:**
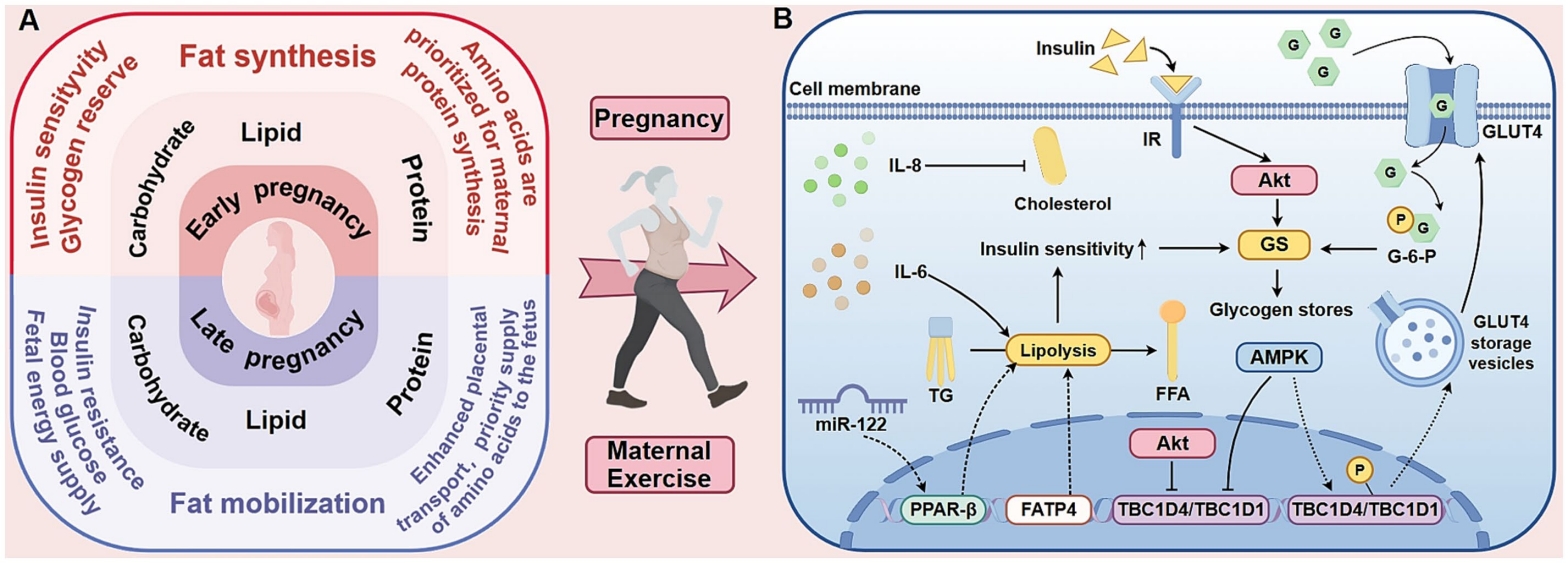
The metabolic characteristics of the three energy-producing nutrients in pregnant women and the influence of exercise on maternal metabolism. The left side of this figure **(A)** compares the metabolic characteristics of the three energy-yielding nutrients in pregnant women during the first and third trimesters. The first trimester is mainly anabolic, facilitating the storage of fat and glycogen. In contrast, the third trimester shifts to a catabolic state to meet the energy demands of the rapidly growing fetus. Throughout pregnancy, protein is preferentially utilized for maternal protein synthesis and fetal development. The mechanistic diagram on the right **(B)** shows the effects of exercise on maternal metabolism, focusing on intracellular glucose transport and insulin sensitivity. Exercise activates AMPK, promoting the translocation of GLUT4 to the cell membrane and enhancing glucose uptake. Although AMPK and insulin both inhibit TBC1D4/TBC1D1 via multi-site phosphorylation, they exert divergent effects on GS activity. Importantly, AMPK-stimulated glucose transport elevates G-6-P levels, which allosterically activate GS and may thereby override AMPK’s direct inhibitory effect. Additionally, exercise modulates lipolysis-related factors, such as IL-6, TG, and FFA, which influence insulin signaling pathways and improve insulin sensitivity. AMPK, 5′-adenosine monophosphate-activated protein kinase; GLUT4, glucose transporter isoform 4; IL-6, interleukin-6; TG, triglyceride; FFA, free fatty acid; miR, microRNA; PPAR-*β*, peroxisome proliferator-activated receptor-beta; FATP4, fatty-acid transporter protein 4; Akt, Serine/threonine kinase B; G, glucose; G-6-P, glucose-6-phosphate; GS, glycogen synthase; IR, insulin receptor; TBC1D4, TBC1 domain family, member 4; TBC1D1, TBC1 domain family, member 1.

Fetal and placental development can lead to increased maternal energy expenditure (23). All the physiological changes during pregnancy are designed to prepare both the mother and her fetus for optimal metabolic energy supply. This preparation ensures that nutrients are made available for transfer to the fetus, facilitating its growth and development. These adjustments are responsive to various physiological variables, which exhibit a markedly wide normal range during pregnancy. Furthermore, these physiological adaptations persist throughout the duration of pregnancy and continue into the post-partum period (24) (Figure 1A).

#### 2.1.1 Maternal glucose metabolism and glucose metabolism disorders

During pregnancy, a mother’s glucose and insulin metabolism undergoes complex adaptive changes to support the development and growth of the fetus. In the early stages, the placenta secretes several key hormones, including human placental lactogen (hPL), estrogen progesterone, and prolactin. These hormones elevate maternal blood glucose by decreasing maternal liver glycogen storage and enhancing glucose release from the liver. In response to increased blood sugar levels pancreatic *β* cells are stimulated to produce greater quantities of insulin (25). As the pregnancy progresses, insulin resistance begins to manifest, indicated by decreased peripheral insulin sensitivity and increased maternal blood glucose levels (26). In the later stages of pregnancy, elevated levels of insulin and hormonal changes result in a reduction of insulin sensitivity in tissues such as muscle and fat, thereby impairing the mother’s ability to utilize glucose effectively (27). The majority of glucose is transferred to the fetus via the placenta, ensuring that the nutritional requirements of the developing fetus are adequately met.

Imbalances in maternal metabolism during pregnancy such as gestational diabetes mellitus (GDM), dyslipidemia, vitamin D deficiency, can lead to significant complications. GDM occurs when pregnancy-induced insulin resistance exceeds physiological limits, resulting in *β*-cell dysfunction and glucose dysregulation. A clinical retrospective analysis has shown that women with GDM face several short-term risks, including a significantly increased likelihood of chronic hypertension within 24 months postpartum (28). In another analysis, Fallatah et al. found that GDM is associated with higher rates of vaginal lacerations and postpartum hemorrhage during delivery, primarily due to elevated fetal macrosomia (29). Long-term consequences for women with GDM have a substantially increased risk of cardiovascular disease (29). Furthermore, children exposed to GDM are more susceptible to overweight and have an increased likelihood of developing type 2 diabetes mellitus (T2DM) later in life (30).

#### 2.1.2 Maternal lipid metabolism and lipid metabolism disorders

Fatty metabolism undergoes significant changes during pregnancy, characterized by lipid synthesis in early pregnancy to lipid mobilization in late pregnancy. In the early stages, the body prioritizes fat synthesis to ensure energy reserves for later use, driven by increased levels of estrogen, progesterone, and insulin, which promote lipid deposition and enhance lipogenesis (31). Additionally, rising levels of progesterone, prolactin, and human placental lactogen (hPL) support maternal hyperphagia (32), further aiding lipid synthesis (5). During the second trimester, elevated insulin levels promote lipogenesis and reduce fatty acid (FA) oxidation, which contributes to maternal fat accumulation. Conversely, late pregnancy is marked by a catabolic state, with increased lipid mobilization resulting from decreased lipoprotein lipase activity (33), leading to higher levels of free fatty acids (FFAs). In the liver, FFAs undergo *β*-oxidation to provide the primary energy source for the mother, while preserving glucose for fetal use (34). Concurrently, some FAs are re-esterified in the liver to form triglycerides (TGs). Consequently, lipid levels in the maternal circulation become significantly elevated during late pregnancy. Clinical research by Brizzi et al. demonstrated a 145% increase in maternal triglycerides and a 58% increase in total cholesterol at this stage (35). Excessive lipid accumulation or metabolic disorders can trigger complications during pregnancy beyond dysglycemia, with clinical cohort studies linking elevated maternal TG levels to and an increased risk of preeclampsia (36), premature birth, and macrosomia (37). Low levels of total cholesterol (TC), TG, and low-density lipoprotein (LDL) may increase the risk of developing small for gestational age (38). Thus, fat metabolism exhibits stage specific characteristics of early pregnancy synthesis reserve and late pregnancy decomposition energy supply.

#### 2.1.3 Maternal protein metabolism and protein metabolism imbalance

Maternal protein metabolism undergoes significant adaptive changes during pregnancy to support the development of fetal tissues and organs. Pregnant women experience a positive nitrogen balance, meeting the physiological needs of both themselves and the fetus. In the early and middle stages of pregnancy, synthetic metabolism is dominant, prioritizing the storage of protein and nitrogen (33). Isotope studies reveal a decrease in maternal plasma amino acid early in pregnancy as the mother favors amino acid synthesis over breakdown (39). This process activates the phosphoinositol 3-kinase /serine threonine kinase/mTOR pathway in the liver, promoting protein synthesis (26). Undoubtedly, it not only meets the needs of fetal development but also strengthens the fetal development and strengthening thighs and hip muscles for natural childbirth. In the later stages of pregnancy, metabolism shifts to a more catabolic state to prioritize fetal needs (33). This is characterized by increased expression of amino acid transporters, facilitating the transfer of amino acids to the fetus. Research from animal models indicates that protein-metabolizing transporters, such as SNAT1 and GLUT3 in the placenta, are upregulated in late pregnancy, enhancing the supply of amino acids (40). In contrast, recent clinical evidence suggests that greater consumption of total and plant-based proteins in pregnant women is linked to improved insulin sensitivity (41). Additionally, increased muscle mass, as measured in human studies using bioelectrical impedance analysis, can improve insulin resistance and help stabilize blood sugar levels, thereby aiding in the prevention and management of diabetes (42). Considerably, low protein levels are related to greater severity of infectious diseases in clinical evidence (43) and can impair uterine artery responses to vasodilators while heightening sensitivity to contraction stimuli (44, 45), reflecting reduced smooth muscle relaxation from preclinical investigations. These findings indicate that protein metabolism and maternal muscle reserve are crucial for the health of both the mother and the fetus.

#### 2.1.4 Maternal vitamin D metabolism and the hazards of its deficiency

The metabolic demands of a developing fetus, alongside the mother’s increased nutritional requirements, are critical during pregnancy (46). A key factor is 1,25-dihydroxyvitamin D [1,25(OH)₂D], the active form of vitamin D, which increases markedly in maternal circulation due to the activation of placental 1α-hydroxylase. These levels can rise to two to three times those in non-pregnant women, enhancing intestinal calcium absorption and supporting fetal skeletal mineralization (47). Vitamin D metabolism is significantly enhanced in active form through placental enzyme activation to support fetal calcium demand. Inadequate 1,25(OH)₂D reserves can increase susceptibility to complications during pregnancy. Research by Wagner et al. suggested that vitamin D supplementation may reduce the risk of preterm birth, asthma, preeclampsia, and gestational diabetes (48). Additionally, Tous et al. have linked maternal vitamin D deficiency to adverse outcomes for infants, including preterm birth and small size for gestational age, potentially due to placental insufficiency (49). Therefore, routine antenatal screening and targeted vitamin D supplementation may serve as effective interventions to prevent these adverse outcomes.

In summary, metabolic disturbances in pregnancy significantly impact maternal and offspring health, highlighting the need for early intervention.

### 2.2 The impact of exercise during pregnancy on pregnant women’s health

Exercise during pregnancy, as a non-pharmacological intervention method, emerges as a low-risk and modifiable lifestyle intervention. Crucially, it not only promotes health in pregnant individuals unaffected by metabolic conditions but also serves as a valuable tool in managing those with gestational metabolic disorders. Exercise prevents glucose and lipid dysregulation and enhances vitamin D metabolism in healthy pregnant women.

As shown in Table 1, it summarizes a range of systematic reviews, meta-analyses, and randomized controlled trials (RCTs) on exercise and metabolic health during pregnancy in 2015–2025. The cumulative evidence indicates that engaging in regular moderate-intensity exercise—such as brisk walking, cycling, dancing, and resistance training (RT), as well as their combinations—significantly reduces the risk of metabolic complications, including GDM, gestational hypertension (GH) and preeclampsia. Numerous RCTs have substantiated that exercise is effective in reducing excessive weight gain among pregnant women, improving blood lipid profiles, increasing vitamin D levels, and decreasing insulin demand in select high-risk populations. Furthermore, for pregnant individuals diagnosed with metabolic abnormalities, exercise interventions have demonstrated notable therapeutic value, including improving fasting and postprandial blood glucose levels, stabilizing blood pressure, and reducing the adverse pregnancy outcomes. It is essential to recognize that there exist variations in the forms, frequency, and intensity of exercise employed in these studies. Additionally, some studies rely on self-reported compliance data from participants, which may affect the accuracy of the results.

**Table 1:** Effects of exercise in women with metabolic diseases during pregnancy.

Metabolic disease	Author/year	Study	Population	Exercise or physical activity intervention	Results
GDM	Cordero et al., 2015 (132)	RCT	*N* = 342 (IG = 101, CG = 156, final analysis 257): Pregnant women in Spain, with an average age of 33.2 ± 4.3 years old; Healthy pregnant women without obstetric contraindications; Starting from 10 to 12 weeks of gestation and continuing until late pregnancy.	50–60 min, 3 times a week, starting from early to late pregnancy; Moderate intensity land and water, AE and RT (including pelvic floor muscle training and relaxation).	Reduced incidence of GDM by 90%.
	Halse et al., 2015 (133)	RCT	*N* = 40 (EX = 20, CON = 20): Pregnant women in Australia with singleton pregnancy; GDM patients; Starting from 28 weeks of pregnancy and continuing for 6 ± 1 weeks.	Five times a week, each lasting 25–45 min, for approximately 6 weeks; Moderate intensity home spinning bikes (supervised) and self-selected AEs (walking, cycling, swimming, yoga, etc., unsupervised).	36% reported improved glucose control.
	Barakat et al., 2018 (134)	RCT	*N* = 456 (EG = 234, CG = 222): Pregnant women in Spain with singleton pregnancy; Healthy pregnant women without risk of diabetes and premature delivery; Following up until 38–39 weeks of pregnancy.	Three times a week, 55–60 min, lasting from 8–10 weeks to 38–39 weeks of pregnancy; Moderate intensity exercise under supervision (warm-up, aerobic dance, light RT, coordination and balance training, stretching, pelvic floor muscle training, and relaxation).	Reduced prevalence of GDM from 6.8 to 2.6% and the proportion of pregnant women with excessive weight gain.
	Rasmussen et al., 2020 (135)	Systematic review and meta-analysis	Healthy pregnant women.	Single 30 min moderate intensity treadmill exercise.	Reducing blood glucose levels in pregnant women in the short term.
			Women at risk of GDM.	Moderate intensity walking or cycling per session.	
	Zhao et al., 2022 (136)	RCT	*N* = 99 (EG = 49, CG = 50): Pregnant women in China with singleton pregnancy; BMI < 40; More than 6-week intervention at 24–31 weeks of gestation.	50–60 min, 3 times a week, lasting until late pregnancy; Moderate intensity resistance exercise (primarily upper/lower limbs, with stretching and relaxation).	Improved fasting and postprandial glycemic levels.
	Martinez-Vizcaino et al., 2023 (137)	Meta-analysis	59 systematic reviews or meta-analyses, including>200 RCTs and N > 270,000: Pregnant women come from 27 countries across five continents; Healthy participants without exercise contraindications, excluding specific diseases and high-risk populations for GDM or HDP; Database construction until August 2021.	≥ 3 times per week; Light to moderate strength; ≥ 30–45 min each time; AE is mainly carried out under supervision, such as walking, cycling, treadmills, and exercise classes.	Reduced the risk of GDM by 39% (OR ≈ 0.61) and the risk of GH by 47%.
	Zhang et al., 2024 (138)	Meta-analysis	*N* = 2,712, 39 RCTs: Pregnant women come from 15 countries (China, United States, Australia, etc.); GDM patients.	30–60 min at least 3 times a week, with interventions covering different stages of pregnancy; Mostly consisting of moderate intensity exercise (AR, structured exercise, postprandial walking, or yoga).	Reduced insulin requirements.
	Allotey et al., 2026 (139)	Individual participant data and network meta-analysis	104RCTs, *N* = 35,993; Trails come from Europe, North America, South America, Australia etc.; White women with an average age of 29 years, 10% of whom have a history of pregnancy diabetes	Water aerobics, fitness sessions or exercise programs, and strength training with varying levels of duration, intensity and timing	Lifestyle interventions reduced the risk of gestational diabetes by 20%, with physical activity-based interventions showing the greatest effectiveness (36% reduction).
Dyslipidemia	Ramírez et al., 2017 (82)	Secondary analysis of RCT	*N* = 67 (EG = 33, CG = 34): Pregnant women in Colombia with low income and Latinx background; Starting from 16 to 20 weeks of pregnancy.	3 times a week, under 60 min of supervision with 12 weeks (from 16–20 weeks of pregnancy to 28–32 weeks) totally; a combination of moderate to high-intensity AE and RT (10 min warm-up walk, 30 min aerobic, 10 min resistance, 10 min relaxation).	Reduced LDL-C and TG aberrant rises.
	McDonald et al., 2021 (52)	Secondary analysis of RCT	Final sample *N* = 77 (exercise = 18, control = 54): Pregnant women in the USA and Spain aged 18–40 years; With normal weight/overweight/Grade I obesity; Starting from 12–16 weeks of pregnancy.	At least 3 times a week with a total duration of over 150 min until delivery; Moderate intensity AE (such as treadmills, spinning bikes, etc.).	Significantly reduced circulating levels of TG, TC, and LDL relative to sedentary controls.
Vitamin D deficiency	Gustafsson et al., 2019 (140)	RCT	*N* = 855 (IG = 429, CG = 426): Pregnant women in Norway; Healthy and low-risk participants; Starting from 18 to 22 weeks of gestation.	3 times a week, 12 weeks; Moderate intensity aerobic + strength training (including 1 supervision+2 home exercises).	Increased circulating concentrations of total 1,25(OH)₂D by 1.9 nmol/L, free 1,25(OH)₂D by 0.55 pmol/L, and bioavailable 1,25(OH)₂D by 0.15 nmol/L.
Hypertension	Spracklen et al., 2016 (141)	Case control study	*N* = 673: Pregnant women in the United States with an average age of approximately 30 years old; Healthy and BMI ≈ 25; Follow up at 6–36 weeks of pregnancy.	At least 8 h per week; Daily activity or increase leisure time (including work, household chores, leisure, and exercise; also assess sedentary time).	Increasing total physical activity can reduce the risk of preeclampsia, while prolonged work or static standing in the workplace can increase the risk of preeclampsia.
	Wang et al., 2025 (142)	Clinical RCT	*N* = 200 (observation group = 110, control group = 90): Pregnant women in Gansu, China; GH; 2023.5–2024.5.	30 min daily, lasting until delivery; Moderate intensity walking combined with systematic care (psychological, dietary, posture, medication guidance).	After intervention, the systolic and diastolic blood pressure of the experimental group were significantly better than those of the control group (both *p* < 0.05).
	Syngelaki et al., 2019 (143)	Systematic review and meta-analysis	*N* = 7,236, 23 RCTs, including 3 investigating the effect of exercise on risk of preeclampsia, 5 investigating the effect of exercise and 1 investigating the effect of diet and exercise.	Exercise intervention	The weight gain of pregnant women in intervention group was prominently lower, which does not affect the risk of pulmonary embolism and hypertension.
Weight gain	Kuang et al., 2023 (144)	Systematic review and meta-analysis	15 RCTs, *N* = 6,812: overweight or obese pregnant women (BMI ≥ 25 kg/m^2^), without serious complications such as diabetes, cardiorenal disease or hypertension.	3–5 times a week, 30–60 min each time, and lasting until late pregnancy (usually around 36 weeks of pregnancy); Moderate intensity AE (such as walking, stationary cycling, AE, etc.) or mixed exercise (AE and RT training).	Reduced weight gain during pregnancy (SMD = −0.20, 95% CI: −0.31 to −0.08, *p* < 0.001).

GDM, Gestational diabetes mellitus; RCT, Randomized controlled trial; GH, Gestational hypertension; AE, Aerobic exercise; RT, Resistance training; BMI, Body mass index; BP, Blood pressure; CG, Control group; EG, Experimental group; EX, Exercise group; IG, Intervention group.

### 2.3 The impact of exercise on maternal metabolism

Exercise enhances metabolic and cellular functions, including insulin sensitivity, glucose and lipid homeostasis, mitochondrial biogenesis, and oxidative capacity, alongside conferring antioxidant and anti-inflammatory effects. These adaptive mechanisms underlie its benefits for pregnant women (Figure 1B).

#### 2.3.1 Improved insulin sensitivity and glucose regulation

Maternal exercise can effectively reduce blood glucose levels by improving insulin sensitivity. Although the benefits of chronic exercise are well-known, most evidence derives from nonpregnant cohorts. Flores-Opazo et al. demonstrated that exercise can activate the glucose transporter isoform 4 (GLUT4) in skeletal muscle to promote muscle glucose uptake and utilization (50). This process is stimulated by 5′-adenosine monophosphate-activated protein kinase (AMPK), which phosphorylates TBC1 domain family, member 1 (TBC1D1) and TBC1D4 to facilitate GLUT4 translocation to the plasma membrane (51). Although the study did not systematically analyze pregnant or postpartum populations, it nevertheless provided evidence supporting the exercise-induced upregulation of Glut4 expression. Additionally, researchers found that aerobic exercise (AE) during pregnancy significantly reduces insulin levels during the later stages and reduces the physiological rise in insulin from mid- to late gestation (52). However, the specific tissue-level mechanisms are still not fully understood.

#### 2.3.2 Improved mitochondrial biogenesis and oxidative capacity

Research on maternal exercise, mitochondrial biogenesis, and gestational oxidative capacity is still currently limited. RT can elevate mitochondrial respiratory capacity and protein expression in human skeletal-muscle to improve mitochondrial function (53). In support, recent studies reported that exercise can stimulate PGC-1α–mediated mitochondrial biogenesis, expanding oxidative capacity and countering the mitochondrial dysfunction associated with insulin resistance and metabolic disorders (54). These adaptations may help mitigate gestational mitochondrial dysfunction and related insulin resistance.

#### 2.3.3 Modulation of inflammatory cytokines

Maternal exercise improves insulin sensitivity by lowering pro-inflammatory cytokines that disrupt insulin signaling. In a clinical cross-sectional study of pregnant women, Korkmazer et al. reported that circulating tumor necrosis factor-alpha (TNF-*α*) at 24–28 weeks gestation serves as an independent predictor of insulin resistance (*r* = 0.49, *p* < 0.05) (55). Further supporting this in a clinical prospective case–control setting, Varthaliti and colleagues demonstrated that elevated interleukin-6 (IL-6) and TNF-α in the first trimester predict later GDM with high sensitivity (56). Importantly, maternal exercise can modulate these inflammatory factors. In a randomized trial, Van Poppel et al. observed that 12 weeks of moderate-to-vigorous prenatal exercise reduced IL-6 by 18% while enhancing first-phase insulin secretion (*β* = −0.36, *p* < 0.01), supporting that IL-6 modulation may mediate the positive effects of exercise on insulin sensitivity (57).

#### 2.3.4 Optimization of lipid metabolism

Maternal exercise is instrumental in addressing pregnancy-related insulin resistance by normalizing lipid metabolism. Elevated triglyceride and FFAs can disturb insulin signaling in the liver and skeletal muscles of obese pregnant women (58). Evidence suggests that in obese rats, exercise can lower FFA levels through the enhancement of *β*-oxidation, mediated via the microRNA-122 (miR-122)/peroxisome proliferator-activated receptor-beta (PPAR-β) signaling axis in preclinical animal models (59). Exercise during pregnancy modulates maternal-fetal circulating cytokines (e.g., IL-1β, IL-6, IL-8, IL-10, TNF-*α*) to influence immunometabolic adaptations, with concurrent exercise training associated with more favorable maternal lipid profiles in the context of elevated IL-8 (60). A separate study on exercise during pregnancy further reported that increased IL-8 levels were correlated with reductions in both total and LDL-cholesterol reflecting in an RCT related humans (60). Moreover, maternal exercise enhances fatty-acid utilization by upregulating placental fatty-acid transporter protein 4 (FATP4) (61). These findings highlight the importance of maternal exercise in optimizing lipid metabolism through the regulation of myokine activity and FA transport.

#### 2.3.5 Attenuation of oxidative stress

Moderate exercise is essential in mitigating gestational metabolic dysfunction by restoring redox balance. Oxidative stress, marked by elevated placental HSP70 and reduced glutamate-cysteine ligase modifier (GCLM), is strongly associated with insulin resistance and adverse pregnancy outcomes (62). Clinically, engaging in moderate-to-vigorous physical activity reverses this trend, resulting in decreased HSP70 and increased GCLM levels, which signifies reduced oxidative damage in the placenta of women with obesity (63). Moreover, moderate exercise leads to an acute rise in systemic and placental Apelin levels (64), which then suppresses NADPH oxidase activity and enhances antioxidant enzyme expression, reducing endothelial oxidative stress and the risk of preeclampsia (65).

Collectively, these mechanisms improve maternal metabolic health by preserving glucose homeostasis, optimizing lipid profiles, and alleviating oxidative stress, ultimately helping to prevent typical pregnancy-related metabolic complications.

### 2.4 Further exploration of exercise during pregnancy

Exercise exerts a beneficial effect on the metabolic health of pregnant women, yet the extent of these positive outcomes varies due to individual characteristics, exercise types, and intensity levels. Roland et al. revealed minimal changes in maternal fat mass and fat-free mass post-exercise interventions, but found a significant inverse link between increased daily step counts and reductions in fat mass, visceral adipose tissue, and body fat percentage. This outcome may be partially attributed to lower adherence rates during mandatory exercise sessions (66). Parallelly, in two other RCTs, Fernández-Buhigas et al. (67) and Xu et al. (68) reported minimal health marker differences and no significant risk reduction, respectively, citing challenges in quantifying pregnancy exercise. Given these complexities, standardized guidelines for optimizing pregnancy exercise are crucial. Pregnant women should follow the FITT principle—frequency, intensity, time, and type of exercise to balance health benefits and safety (69). Table 2 summarizes recent prenatal exercise studies by the FITT principle, highlighting the need for further research to refine exercise protocols for pregnant women.

**Table 2:** Prenatal exercise intervention based on FITT principle.

Author/year	Study	Population	Exercise intervention (FITT)				Results
			Frequency	Intensity	Time	Type	
Roland et al. (2024) (66)	Secondary analysis of RCT	*n* = 150: Healthy, inactive pregnant women with an average age of 31 years and gestational age<15 weeks; Measure body composition at 28 weeks of pregnancy and after delivery	3 times a week	Moderate intensity	1 h each time	Supervised exercise training conducted in the gym and swimming pool. Simultaneously monitor the number of steps taken	Higher step counts correlated with reductions in fat mass, visceral adipose tissue, and body fat percentage
Fernández-Buhigas et al. (2020) (67)	Secondary analysis of RCT	*n* = 92: Healthy pregnant women with an average age of 33 years, gestational age<16 weeks, and irregular exercise exceeding 30 min (3 days a week); 2014–2015	3 times a week	Mild to moderate	Each class lasts for more than 30 min, including 10 min of warm-up, followed by 25 min of cardiovascular exercise, 10 min of strength training, 5 min of coordination and balance training, 5 min of pelvic floor exercises, and finally 5 min of stretching and relaxation.		Minimal difference in cardiovascular, metabolic, and renal outcomes between exercise and non-exercise groups
Xu et al. (2024) (68)	Systematic review and meta-analysis	37 RCTs, *n* = 10,699: Healthy pregnant women, GDM patients, or women with a history of GDM.	Mainly moderate frequency exercise during pregnancy and postpartum period	Mainly low to moderate, adjusted according to individual health status and pregnancy stage	The duration of exercise varies, but typical interventions involve exercising for about 30 min multiple times a week	Usually includes a combination of aerobic exercise and resistance training	Although the risk of GDM is reduced after exercise, and the blood sugar of GDM patients is improved, exercise has no significant effect on the incidence rate of T2DM after GDM
Susanti et al. (2024) (70)	Quasi-experimental study	*n* = 15: Healthy pregnant women; Exercise intervention lasts for 4 weeks	Once a week lasts for a total of 4 weeks	Mild	1 h per session	-	Improved mood and reduced anxiety
Bennett et al. (2023) (72)	Systematic review and meta-analysis	20 RCTs, *n* = 6,732: Pregnant women at risk for GDM	-	Suggested moderate intensity, i.e., based on MET value (metabolic equivalent), it is necessary to reach 600 MET · min·wk.^−1^ per week	At least 150 min of moderate intensity exercise per week	Covering different forms of aerobic exercise and strength training (such as walking, cycling, swimming, etc.)	To lower the risk of GDM, pregnant women should exceed a specified exercise threshold of 600 MET·min·wk.^−1^
Watkins et al. (2021) (74)	Secondary analysis of prospective cohort studies	*n* = 811: Healthy pregnant women with gestational age<20 weeks;	A KPAS score greater than 10.8 indicates a high level of physical activity, otherwise it indicates a low level of physical activity			Sports (such as walking, running, swimming, and ball games), work, daily habits, household chores (such as cooking, cleaning, sweeping, etc.)	Higher levels of physical activity in late pregnancy are associated with shorter active delivery times and a reduced likelihood of prolonged first stage labor
Wowdzia et al. (2023) (75)	RCT	*n* = 15: Healthy pregnant women with an average gestational age of 33 years and an average gestational age of 27 weeks; Exercise intervention lasts for 4 weeks	Each person only did one HIIT and one MICT	HITT and MICT	The total duration of HIIT is about 19 min; The total duration of MICT is 30 min	Cycle ergometer	Both single HITT and MICT are safe for healthy pregnant women
Catov et al. (2018) (76)	Prospective Cohort Study	*n* = 10,038: Healthy pregnant women with gestational age<6 weeks and single pregnancy; Starting from the 6th week of pregnancy and ending around the 30th week of pregnancy	The proportion of women with high exercise frequency is gradually decreasing. Only a few pregnant women have reached three times a week.	The exercise intensity of most participants is moderate, with a weekly activity intensity of approximately 3 to 6 METs	Pregnant women’s exercise time mainly focuses on 150 min of moderate intensity activity or at least 75 min of high-intensity activity per week	The most common ones are jogging, walking, aerobic exercise, and yoga	Significantly reduced the risk of GDM and childbirth complications
Chen et al. (2024) (77)	Prospective RCT	*n* = 40: Pregnant women with gestational hypertension; 2023–2024	3–5 times a week	Moderate intensity: Target heart rate of 100–120 bpm	45–65 min each time	AE (warm up + moderate intensity walking + relaxation), and RT (warm up + sitting dumbbell practice + relaxation)	Improved physical exercise levels, sleep quality, blood pressure, blood lipids, and pregnancy outcomes
Wang et al. (2025) (78)	RCT	*n* = 46: Pregnant women with gestational age less than 6 weeks and single pregnancy who are overweight or obese; Starting from week 16 and continuing until week 36 of pregnancy	3 times a week	Moderate intensity (40–59% V_O2_peak)	1 h each time	AE (warm up + treadmills, elliptical machines, horizontal bicycles, rowing boats + stretching exercises) and RT (warm up + sitting posture machine, dumbbell, resistance band/dumbbell, fitness ball, bench and/or mat + stretching exercises)	Better blood glucose control and cardiovascular function compared to AE or RT alone
Claiborne et al. (2025) (79)	Secondary analysis of data from three blinded, prospective RCT	*n* = 176: Pregnant women who are healthy or overweight and have a gestational age of less than 16 weeks; Starting from 12–16 weeks of pregnancy and continuing until 37–40 weeks of pregnancy	3 times a week	Moderate strength	50 min per week	AE (warm up + treadmills, elliptical machines, horizontal bicycles, rowing boats + stretching exercises) and RT (warm up + sitting posture machine, dumbbell, resistance band/dumbbell, fitness ball, bench and/or mat + stretching exercises)	Effectively reduced resting BMI-associated lactate elevation during pregnancy

RCT, Randomized controlled trial; AE, Aerobic exercise; RT, Resistance training; GDM, Gestational diabetes mellitus; GH, Gestational hypertension; T2DM, Type 2 diabetes mellitus; HIIT, High-intensity interval training; MICT, Moderate-intensity continuous training; METs, Metabolic equivalent of tasks; BMI, Body mass index.

#### 2.4.1 The frequency of exercise during pregnancy

The frequency of exercise during pregnancy significantly impacts the effectiveness of health interventions. While low-frequency exercise is better than none, it yields limited benefits. Clinical research by Susanti et al. indicates that low-frequency, mild exercise (1 h/session, 1session/week, 4 weeks) can improve mood and reduce anxiety in pregnant women (70). Moderate-intensity activity, typically defined as activity during which one can talk but not sing, is recommended during pregnancy. It has been associated with reduced the risks of pregnancy-related complications, including GH, GDM, cesarean sections and instrument-assisted delivery (71). Examples of such activities include brisk walking, water aerobics, stationary cycling at moderate effort, resistance training, carrying moderate loads, and household tasks such as gardening or window cleaning.

#### 2.4.2 The intensity of exercise during pregnancy

Exercise intensity is crucial for improving pregnancy outcomes. Although low-intensity exercise is popular among pregnant women, its effectiveness may be limited. Bennett et al. found that to significantly lower the risk of GDM, pregnant women should exceed a specified exercise threshold as over 600 MET·min·wk.^−1^ (72). The American Heart Association (AHA) and the WHO recommend at least 150 min of moderate-intensity AE weekly (73). Higher physical activity levels in late pregnancy are associated with shorter duration of active delivery (74). While high-intensity exercise is rarely chosen, Wowdzia et al. demonstrated that both high-intensity intervals and sustained moderate-intensity exercise are safe for healthy pregnant women (75). Therefore, personalized adjustments of exercise intensity are essential.

#### 2.4.3 The timing of exercise during pregnancy

The timing of exercise interventions is a crucial factor for effectiveness. Initiating exercise early in pregnancy is more beneficial than starting mid or late. Bennett et al. found that early intervention can significantly reduce the risk of GDM, while exercise after mid-pregnancy has no significant effects (72). Additionally, engaging in moderate to high-intensity exercise for over 300 min per week in late pregnancy can decrease childbirth complications (69). In terms of controlling the duration of a single exercise, by observing five different maternal exercise human models, Catov et al. found that conducting moderate and high intensity exercise for more than 30 min daily during pregnancy could significantly reduce the risk of GDM (76), underscoring the importance of exercise duration.

#### 2.4.4 The types of exercise during pregnancy

Researchers have assessed appropriate exercise modalities for pregnant women by comparing AE and RT. Evidence suggests that AE and RT are a more effective intervention strategy. Chen et al. reported that AE and RT significantly improved physical activity levels, sleep quality, blood pressure, blood lipids, and pregnancy outcomes in patients with GH (77). Wang et al. found that a combination of AE and RT leads to better blood glucose control and cardiovascular function compared to AE or RT alone (78). Meanwhile, Claiborne et al. found that AE, particularly when combined with RT, effectively reduces resting lactate elevation during pregnancy, especially in overweight women (79). In addition to the aforementioned human research, animal studies, such as those conducted by Stevanović-Silva et al., showed that moderate-intensity AE positively affects liver metabolism in pregnant mice (80). The combination of AE and RT provides substantial benefits for metabolic health, hemodynamics, and pregnancy outcomes, particularly for high-risk women. However, the effects of exercise during pregnancy are influenced by factors such as intervention timing, exercise type, frequency, intensity, and maternal baseline health. Future research should clarify the dose–response relationship of exercise, determine optimal regimens for different groups of pregnant women, and enhance studies on long-term maternal and infant outcomes to create personalized and precise prenatal exercise guidelines.

## 3 Exercise during pregnancy and metabolic health of offspring

**Figure 2:**
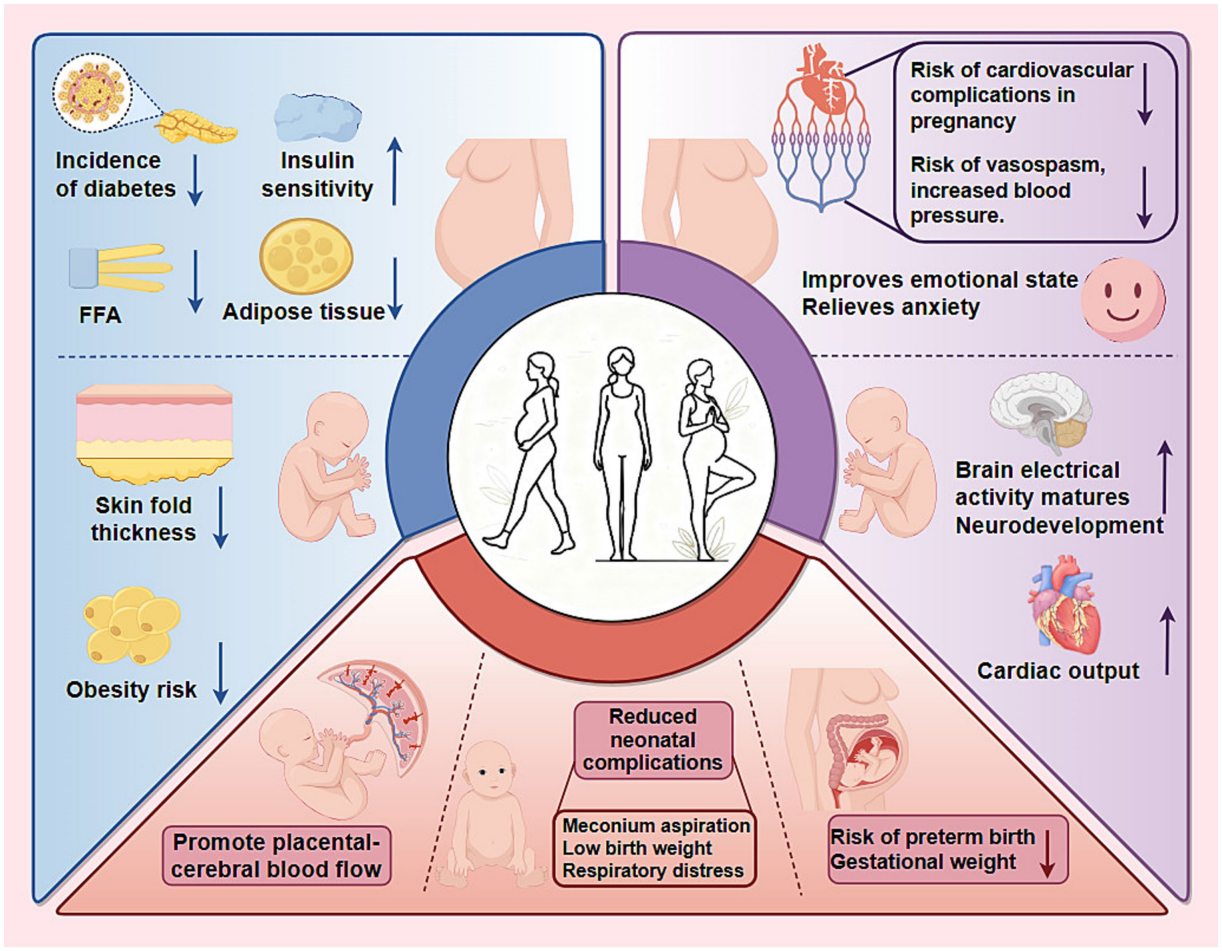
Benefits of exercise during pregnancy for pregnant women and their offspring. Engaging in exercise during pregnancy offers significant health advantages for both mothers and their children, categorized as follows: 1. Molecular and metabolic effects: Exercise improves maternal insulin sensitivity and reduces FFA levels, lowering the risk of gestational diabetes. This metabolic shift also reduces subscapular skin fold thickness in offspring, thereby decreasing their long-term obesity risk. 2. Organ system effects: Regular exercise reduces the mother’s risk of cardiovascular issues and hypertension, while improving her emotional well-being. In fetuses, exercise promotes increased cardiac output and accelerates neurodevelopment. 3. Pregnancy outcomes: Exercise aids in regulating gestational weight and enhances placental and cerebral blood flow, significantly reducing the risk of preterm birth and neonatal complications, such as meconium aspiration, respiratory distress, and low birth weight.

Exercise during pregnancy is not only beneficial for maternal health but also exerts a profound, long-lasting effect on the metabolic health of offspring (Figure 2). The underlying mechanisms involve epigenetic remodeling, placental signaling, regulation of maternal metabolic status, and mitochondrial reprogramming. In addition, exercise during pregnancy also has positive impacts on other physiological systems of offspring, including the cardiovascular, nervous, and motor systems.

### 3.1 The impact of exercise during pregnancy on the metabolic health of offspring

#### 3.1.1 Exercise and positive effects on pregnancy outcomes

To assess the impact of maternal exercise during pregnancy on fetal safety, Makaruk et al. monitored the cerebroplacental ratio in pregnant women involved in a structured exercise program. Their findings indicated that exercise promoted placental-cerebral blood flow (81). Notably, for pregnant women with normal pre-pregnancy weight, regular exercise is associated with a reduction in complications such as meconium aspiration, cyanosis, and respiratory distress in newborns (82). Chen et al. also found that exercise reduced the risk of premature birth by 15% and decreased the risk of being small for gestational age and large for gestational age by 17% (83). For obese pregnant women, exercise also reduced the chances of premature birth and low birth weight (83). Haakstad et al. reported that exercising twice or more per week is linked to a 73% reduction in gestational weight gain and the risk of macrosomia in women with advanced maternal age (84). These findings challenge the misconception that exercise during pregnancy is harmful.

#### 3.1.2 Exercise on glucose metabolism in offspring

Maternal exercise during pregnancy improves maternal glycemic control, attenuates GDM incidence, and programs superior glucose homeostasis in offspring. By enhancing maternal insulin sensitivity, exercise interrupts the intergenerational transmission of metabolic dysfunction, curbing the heightened risk of obesity, insulin resistance and T2DM historically associated with GDM. Animal experiments corroborate these benefits: low intensity, on a rodent treadmill at 30% VO_2_Max/30-min/session/3x/week exercise in dams prevents glucose tolerance and hyperinsulinemia in offspring subjected to early post-natal overnutrition (85); programmed wheel-running in high-fat and high sugar (HFHS) fed dams normalized offspring glycaemia and improved glucose tolerance (86); housing female dams on a high-fat diet in cages with running wheels completely reversed impaired glucose tolerance and significantly reduced fasting insulin in female offspring (87).

Pioneeringly, Carter et al. wonder whether perinatal exercise, a non-pharmacological intervention, can improve the metabolic health of offspring. In this experiment, female mice in the exercise cohorts were able to voluntarily use running wheels throughout pregnancy and postpartum. They found that the offspring of the exercise group showed an overall increase in glucose sensitivity, a decrease in blood glucose concentration, and a decrease in fat mass percentage in male offspring (88). Similarly, the research by Stanford et al. aims to explore whether maternal exercise can counteract the adverse effects of maternal obesity on offspring metabolic health. In particular, they observed that offspring of exercising mothers exhibited a lower rate of glucose clearance *in vivo*, which reflects an improvement in glucose homeostasis in the offspring (89). Different from previous studies that primarily focused on maternal exercise, Alves-Wagner et al. have been continuously exploring whether grandmaternal voluntary wheel running (F0 generation) during pregnancy can improve metabolic health in F2 offspring. They found that wheel running improved glucose tolerance and insulin resistance, particularly in male F2 offspring, with the benefits transmitted through F1 males and females, demonstrating a cross-generational effect on metabolism (90, 91). Collectively, these findings establish maternal exercise as a potent, non-pharmacological intervention that safeguards both maternal and progeny metabolic health across generations.

#### 3.1.3 Exercise on obesity-related metabolism in offspring

The impact of prenatal exercise on offspring adiposity is dose-dependent and moderated by maternal metabolic status. Recent meta-analyses indicate that guideline-concordant exercise (at least 150 min/week moderate intensity) initiated before 14 weeks confers modest but consistent reductions in offspring obesity risk, particularly in metabolically healthy mothers.

Human cohorts demonstrate consistent dose-dependent benefits: infants of active mother exhibit reduced subscapular skinfold thickness (92); children whose mothers followed a simple antenatal exercise protocol display a 53% lower obesity risk (83); and at 8 years, any mild maternal exercise lowers overweight/obesity odds by 23%, whereas prolonged sitting raised them (93). These advantages persist into adulthood, with maternal exercise reducing fat mass and enhancing pyruvate and lipid handling even under post-natal high-fat feeding in rats (94). Other experiments in animals have also supported these benefits. Offspring born to exercised dams show lower body weight and adiposity across the lifespan (85). Hepatic interrogations reveal that maternal exercise normalizes TG accumulation and reversed HFHS feeding-induced elevations in long-chain acylcarnitine by augmenting mitochondrial respiratory capacity, thereby mitigating non-alcoholic fatty liver disease risk (95).

However, several RCTs have reported marginal or no effects. The individual participant data meta-analysis of RCTs found no sustained anthropometric benefits in 3–5-year-old children of overweight/obese women despite lifestyle interventions (96), with approximately 30% remaining at high BMI risk. Similarly, 150 min/week moderate exercise failed to improve offspring metabolic markers in the original Finnish gestational diabetes prevention study (RADIEL), though exploratory analyses suggested benefits in GDM subgroups (97). Tanvig et al. showed that a lifestyle intervention involving daily moderate-intensity physical activity for 30 to 60 min, initiated at 10–14 weeks of gestation in obese pregnant women, resulted in no significant differences in their offspring’s BMI Z-scores, abdominal circumference, or metabolic risk factors such as glucose, insulin, HDL, or triglycerides (98). The multicenter RCT also showed no differences in overweight and obese women on cord blood cardiometabolic and inflammatory biomarker outcomes after following lifestyle advice (99).

In summary, appropriately dosed antenatal exercise (meeting guideline thresholds of at least 150 min/week moderate intensity) is a safe, low-cost intervention that durably improves offspring lipid metabolism and reduces adiposity risk, though efficacy is contingent on maternal metabolic status, intervention timing, and adherence.

### 3.2 The mechanism of the impact of exercise during pregnancy on the metabolic health of offspring

**Figure 3:**
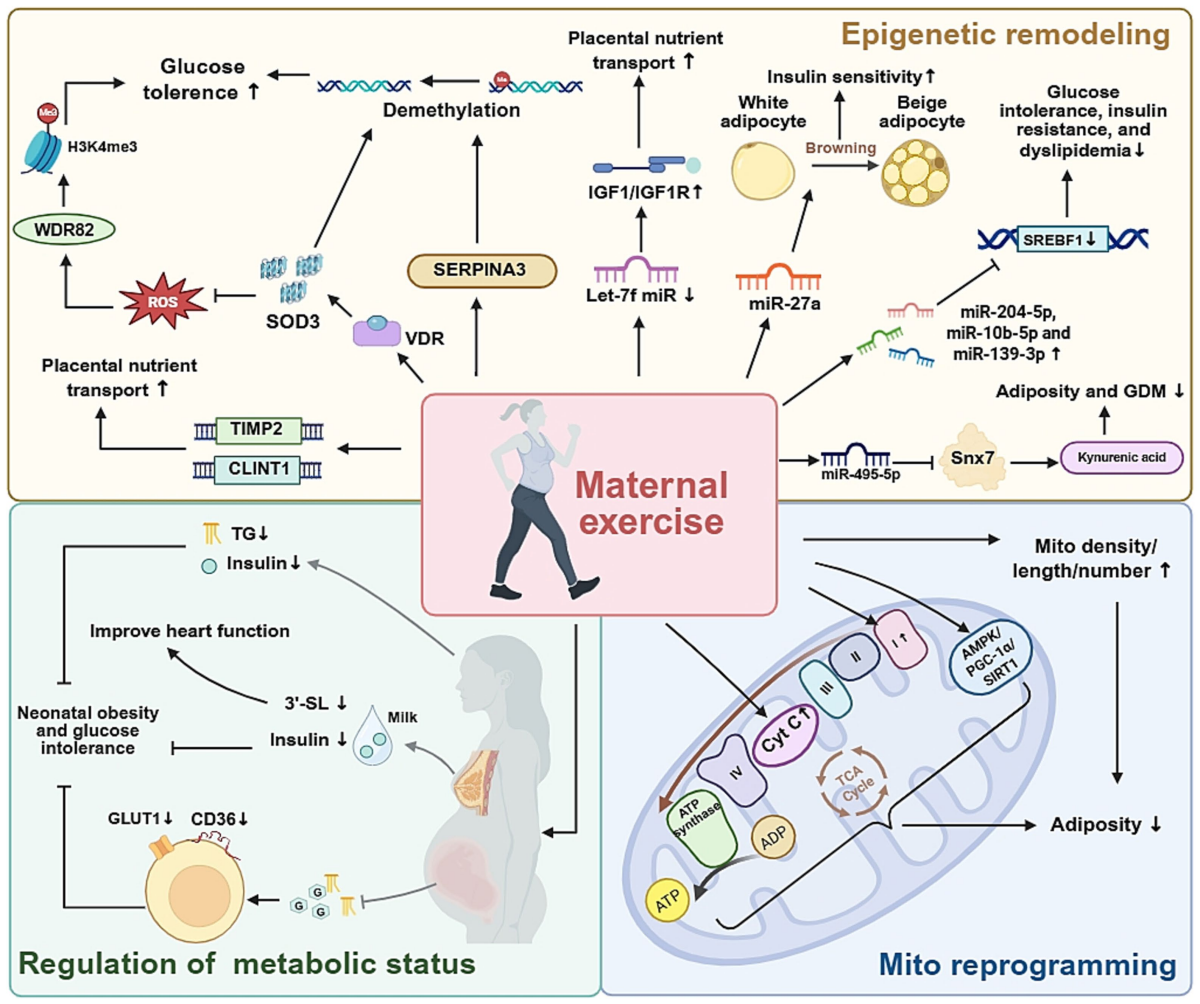
The molecular and metabolic pathways involved in maternal exercise, and their effects on offspring health.

Plenty of evidence attributes the metabolic benefits of maternal exercise to non-genomic programming. Parallel investigations impute additional roles to maternal metabolic milieu and fetal mitochondrial biogenesis, collectively shaping lifelong metabolic trajectories (Figure 3).

#### 3.2.1 Maternal exercise during pregnancy regulates fetal development through epigenetic mechanisms

The epigenetic mechanism role in mediating the impact of maternal exercise during pregnancy on the metabolic health of offspring has garnered significant attention. Among various epigenetic regulatory mechanisms, changes in placental DNA methylation play a central role by regulating gene expression and metabolic function. Previous clinical studies have found that increased SERPINA3 expression is associated with reduced methylation in its 5′ region in cases of preeclampsia and fetal growth restriction (100), suggesting that SERPINA3 family members may be crucial for fetal development.

To further elucidate the role of SERPINA3, Li et al. performed functional experiments by silencing and overexpressing SERPINA3 in cellular models (101), finding that maternal exercise promotes demethylation of the Klf4 gene promoter in fetal preadipocytes through regulation of SERPINA3C protein, thereby inhibiting adipose inflammation and improving glucose tolerance (92). Beyond SERPINA3, Zhao et al. used genome-wide methylation scans to identify TIMP2 and CLINT1 as genes influenced by maternal exercise, affecting placental angiogenesis and fetal substance transport (102). These findings highlight the multiple DNA methylation–mediated pathways through which maternal exercise can influence fetal development.

Building on these insights, Kusuyama et al. reported that maternal exercise activates placental vitamin D receptor signaling, increasing superoxide dismutase 3 (SOD3) secretion. This, in turn, promotes DNA demethylation of key metabolic genes, improving offspring liver glucose homeostasis (103). The same team later demonstrated that SOD3 induced by maternal exercise also counteracts the negative effects of a maternal high-fat diet. Specifically, SOD3 stabilized H3K4me3 and protected WDR82 from carbonylation, thus preserving histone-mediated epigenetic regulation of metabolic genes in offspring (104). Notably, recent studies by Xu et al. expanded SOD3’s role beyond metabolism to maternal behavior. Using placenta-specific SOD3 knockout mice, they found that loss of SOD3 impaired nesting and pup-retrieving behaviors, along with reduced serum and pituitary prolactin levels. Mechanistically, SOD3 deficiency disrupted FGF/FGFR signaling by suppressing TET/IDH/*α*-ketoglutarate–dependent DNA demethylation at Fgf1 and Fgfr2 promoters, downregulating prolactin expression (105). These findings collectively highlight DNA methylation as a key epigenetic mechanism through which maternal exercise influences fetal development and metabolic health.

Beyond SOD3, accumulating evidence indicates that other exercise-sensitive epigenetic pathways also play a crucial role in shaping offspring development. In particular, recent preclinical studies have identified several microRNAs (miRNAs) that serve as links between maternal exercise and offspring health outcomes. During pregnancy, maternal exercise promotes placental nutrient transport by downregulating Let-7f miRNA expression while specifically upregulating IGF1/IGF1R protein expression in the placenta. Reduced placental IGF expression is closely associated with fetal growth restriction (106). Similarly, focusing on placental metabolism, Li et al. investigated the mechanisms by which maternal exercise during pregnancy mitigates the adverse effects of an intrauterine environment in female mice. They reported that maternal exercise reprograms placental miR-495-5p–mediated Snx7 expression and modulates the kynurenic acid metabolic pathway in the context of a prenatal high-fat diet, highlighting the role of miRNA in connecting maternal lifestyle choices to placental metabolism (107). Beyond focusing on the placenta, by establishing a high-fat diet induced obese mouse model, Wang et al. reported that exercise induced extracellular vesicle miR-27a contributes to browning of white adipose tissue and enhances skeletal muscle insulin sensitivity, indicating that circulating miRNAs act as systemic mediators of the beneficial effects of maternal exercise (108). Extending these findings, Zhou et al. demonstrated that maternal exercise programs hepatic miRNA expression in adult offspring, providing long-term metabolic protection. In a mouse model of maternal high-fat diet, voluntary wheel running before and during pregnancy rescued glucose intolerance, insulin resistance, and dyslipidemia in 24-week-old male offspring. Transcriptomic analysis revealed that maternal exercise reversed diet-induced dysregulation of hepatic genes involved in glucose and lipid metabolism, while normalizing three key hepatic miRNAs whose targets regulate cholesterol biosynthesis and epigenetic modification (109). These findings indicate that maternal exercise supports fetal growth by ensuring an adequate nutrient supply through molecular adaptations in the placenta and systemic metabolic improvements mediated by circulating miRNAs.

#### 3.2.2 Maternal exercise during pregnancy regulates fetal development through metabolic adaptations

Regular physical activity initiated either before or during pregnancy has recognized as a powerful metabolic intervention that improves maternal insulin sensitivity and alters the biochemical environment to which the fetus is exposed. These maternal adaptations are mediated through systemic changes in maternal serum metabolites that cross the placenta, as well as through lactarane signaling, which involves alterations in milk composition after birth.

In both human cohorts and rodent models, moderate-intensity exercise has been shown to lower maternal fasting insulin, TGs and leptin levels, while increasing adiponectin and the insulin-sensitive myokine irisin. Vega et al. demonstrated that obese female rat’s voluntary running for 6 weeks before mating and throughout gestation exhibited lower maternal insulin (−18%) and TG levels (−15%), along with improved HOMA-IR (110). These systemic changes were accompanied by reduced expression of placental GLUT1 and CD36, effectively attenuating placental glucose and fatty-acid transport, thereby preventing excessive fetal adiposity (111).

Harris et al. extended their research to the postpartum stage and found that mothers who exercise can secrete milk with higher levels of oligosaccharides 3′- sialyllactose (3’-SL). And 3’-SL is beneficial for the heart function of mouse offspring (112). Further studies revealed that these early-life adaptations translated into long-lasting protection against diet-induced obesity and glucose intolerance. Quiclet et al. (94) reported that offspring of exercised dams displayed improved glucose tolerance and enhanced pancreatic *β*-cell function when challenged with a high-fat/high-sucrose diet at 6 months of age. Importantly, this protective phenotype was replicated when sedentary dams received milk from exercised donors, underscoring the causal role of lactarane factors. Recently, Lu et al. also confirmed the same viewpoint and put forward that maternal exercise can reduce insulin levels in human maternal breast milk, thereby reducing obesity in offspring (113).

Collectively, these findings demonstrate that maternal exercise optimizes fetal metabolic programming through coordinated prenatal and postnatal pathways, offering a promising strategy for improving offspring metabolic health.

#### 3.2.3 Maternal exercise during pregnancy improves offspring metabolism through mitochondrial programming

Exercise during pregnancy significantly influences the mitochondrial function and energy metabolism of offspring. Mice experiments have shown that exercise during pregnancy can increase the volume density, length, and quantity of mitochondria in offspring skeletal muscle, while also enhancing the activity of key mitochondrial enzymes such as citrate synthase and cytochrome c oxidase (114). To assess whether these findings from animal models are applicable to humans, Jevtovic et al. performed mitochondrial function assays and molecular profiling on human neonatal umbilical cord–derived mesenchymal stem cells (MSCs) (115, 116). They found that maternal exercise during pregnancy reprograms fetal MSCs to achieve greater metabolic efficiency. Specifically, while mitochondrial respiratory capacity is reduced, FA oxidation is enhanced, which helps limit lipid accumulation and lower neonatal adiposity (106). Mechanistic exploration revealed that prenatal exercise activates the AMPK/PGC-1α/SIRT1 axis, optimizes mitochondrial respiration and fat oxidation capacity of infant MSCs, enhancing insulin sensitivity, and ultimately reducing the risk of obesity in infancy (116). Similarly, in clinical studies examining the metabolic phenotype of MSCs, Chaves et al. first discovered that maternal motor specific upregulation of mitochondrial complex I in offspring cells, providing a molecular mechanism for enhancing glucose oxidation ability (117).

In summary, exercise during pregnancy exerts a multifaceted impact on offspring metabolic health through epigenetic remodeling, placental signaling, modulation of maternal metabolic status and milk composition, as well as mitochondrial reprogramming.

### 3.3 Participation of other systems

While current evidence has highlighted the metabolic benefits of maternal exercise for offspring, there is a growing focus on how these changes extend to other organ systems. Notably, the cardiovascular and nervous systems, which are tightly linked to metabolic regulation and long-term health outcomes.

#### 3.3.1 Cardiovascular system

Prenatal exercise exerts beneficial effects on offspring cardiac function through metabolic and epigenetic programming, though effect sizes vary by maternal BMI and exercise dose. Maternal exercise meeting guideline recommendations is associated with improved neonatal cardiac outcomes. A 2023 pilot clinical study demonstrated that supervised AE reduced resting heart rate and increased cardiac output in 1-month-old infants, with more pronounced benefits in offspring of overweight mothers with metabolic abnormalities (118). However, long-term follow-up data in humans remain limited, and these preliminary findings require validation in larger, adequately powered trials.

Translational research provides stronger evidence for more persistent benefits. Maternal exercise in obese mouse models normalized adult offspring heart weight, myocyte area, and ejection fraction, mitigating obesity-induced cardiac hypertrophy (119). Félix-Soriano et al. sought to understand the underlying mechanism and proposed that the cardiovascular health of offspring is primarily associated with factors such as placental release of SOD3 following maternal exercise, regulation of apelin, and the presence of 3’-SL in breast milk (120). Additionally, Cochrane et al. reviewed that exercise attenuates maternal obesity-associated sympathetic hyper-reactivity, mitochondrial dysfunction, and cardiac miRNA epigenetic dysregulation in offspring (121).

Given that a considerable percentage of women enter pregnancy overweight or obese (122), targeted interventions are critical. A recent meta-analysis of RCTs indicated that exercise reduces cardiac remodeling in offspring, but heterogeneity in intervention timing and adherence confounds results (123). Collectively, current evidence suggests prenatal exercise benefits offspring cardiac function, particularly in metabolically compromised pregnancies, but long-term human data remain limited.

#### 3.3.2 Nervous system

Research has also focused on the nervous system, given its central role in cognition, behavior, and emotional regulation, and its sensitivity to intrauterine environmental changes. In human studies, structural and functional outcomes have been explored. Labonte-Lemoyne et al. showed that exercise during pregnancy can promote the maturation of neonatal brain electrical activity, as indicated by lower auditory mismatch response (MMR) amplitudes, a marker of neural development (124). Regarding long-term functional outcomes, Leão et al. found no effects of maternal exercise on language or cognition in offspring at ages 2 and 4, suggesting any potential benefits may be indirectly mediated by factors such as enhanced placental function (125). Nevertheless, the absence of long-term follow-up outcomes leaves these findings inconclusive. In addition to these developmental indicators, clinical studies have also investigated the potential protective role of maternal exercise. Prenatal exercise has also been linked to reduced negative emotionality and faster recovery in infants, potentially through lowering maternal cortisol exposure and altering hippocampal glucocorticoid receptor methylation, thereby enhancing HPA axis regulation (126). Exercise during pregnancy not only contributes to the maturation and functional optimization of the fetal nervous system, but may also provide lasting protection for offspring neural development by improving the maternal environment.

#### 3.3.3 Other systems

Research about maternal exercise on other physiological systems, has revealed its potential to influence metabolic status and foster fetal growth and development. Fetal skeletal muscle development is particularly crucial, as muscle fiber formation occurs primarily during embryogenesis and significantly impacts offspring metabolic health (127). Gao et al. demonstrated that maternal treadmill exercise elevates thyroid hormone levels in both maternal circulation and mouse embryos, enhancing thyroid hormone receptor *α* signaling and upregulating myogenic genes (Pax3, Pax7, Myf5, Myod) through increased promoter binding, thereby promoting embryonic myogenesis (128). These systemic benefits extend to immune regulation; exercise-induced improvements in maternal glycemia and oxidative stress reduce GDM risk and associated fetal immune complications (129). However, immunomodulation by maternal exercise remains an emerging field. While moderate-vigorous activity alters maternal cytokine profiles during pregnancy (130), long-term effects on offspring immunity and the role of exerkines in maternal-fetal immune communication require further investigation (131). Collectively, these findings indicate that maternal exercise fosters a more favorable intrauterine environment, supporting coordinated development of offspring muscular, metabolic, and immune systems.

In summary, maternal exercise exerts broad benefits that extend metabolic improvements, encompassing the cardiovascular, nervous, motor and immune systems.

## 4 Conclusion and future perspectives

Regular exercise is an important therapeutic intervention for addressing obesity and improving metabolic health in the general population. However, the effects of maternal exercise during pregnancy on the metabolic health of mothers and their offspring remain poorly understood, with limited human studies available. Research utilizing rodent models has demonstrated that maternal exercise can enhance the metabolic health of offspring. The accurate evaluation of exercise data, the standardization of questionnaires, and the precise conversion to metabolic equivalent of tasks collectively determine the validity of the information obtained. However, several fundamental questions remain unresolved. Firstly, which specific gestational windows most effectively program lifelong metabolic outcomes in offspring? Emerging evidence suggests that timing may be a critical determinant, yet optimal periods have not been conclusively identified. Secondly, what intensity levels produce benefits versus potential stress, and how do these change during pregnancy? Thirdly, what predominant molecular mechanisms—such as placental adaptations or epigenetic modifications—transmit and maintain the advantages of maternal exercise into adulthood?

Addressing these gaps necessitates a multi-pronged research agenda. Future research should prioritize determining the optimal timing and intensity of maternal exercise to maximize long-term metabolic benefits for offspring. While evidence supports exercise during gestation and lactation, the underlying mechanisms remain insufficiently characterized. Investigating placental exerkines, microRNAs, and mitochondrial adaptations may further elucidate the intergenerational impacts of maternal exercise. In summary, well-designed human studies are essential to validate findings from animal models and create evidence-based guidelines for maternal exercise. Such guidelines will help maximize intergenerational metabolic health benefits, ultimately contributing to improved health outcomes for both mothers and their offspring.

The body of evidence presented in this review underscores maternal exercise as a potent, modifiable intervention with profound implications for transgenerational metabolic health. With obesity and metabolic diseases reaching epidemic proportions globally, maternal exercise offers a unique opportunity for primary prevention. However, its integration into clinical practice demands evidence-based protocols validated in diverse populations. Key tasks include: (1) define critical windows regarding nutrient partitioning and energy metabolism to balance adaptive responses against potential overstress; (2) dissect molecular mechanisms of intergenerational transmission to develop maternal baseline health status, gestational complications, or fetal responsiveness; (3) clarify strategies for sustaining metabolic health across the lifespan, leveraging artificial intelligence (AI) and digital health tools, to advocate for policy changes that promote prenatal exercise as a standard of care. To fully realize the full transgenerational metabolic benefits of maternal exercise demands a globally coordinated research agenda that integrates advanced mechanistic studies with population-specific translational trials. This approach will help us develop clear, personalized guidelines that improve the metabolic health of mothers and their babies while addressing important issues such as dosage, timing, and fairness. To make this happen, we require cohesive policy action across multiple disciplines to place a higher priority on exercise in prenatal care. This will help ensure that scientific discoveries lead to lasting health improvements for communities across generations.
